# *Antarctophthirus microchir* infestation in synanthropic South American sea lion (*Otaria flavescens*) males diagnosed by a novel non-invasive method

**DOI:** 10.1007/s00436-019-06273-2

**Published:** 2019-03-14

**Authors:** David Ebmer, Maria José Navarrete, Pamela Muñoz, Luis Miguel Flores, Ulrich Gärtner, Anja Taubert, Carlos Hermosilla

**Affiliations:** 1 Institute of Parasitology, Biomedical Research Center Seltersberg (BFS), Justus Liebig University Giessen, Schubertstr. 81, 35392 Giessen, Germany; 2 Instituto de Patología Animal, Facultad de Ciencias Veterinarias, Universidad Austral de Chile, Valdivia, Chile; 3 Institute of Anatomy and Cell Biology, Justus Liebig University Giessen, Aulweg 123, 35385 Giessen, Germany

**Keywords:** Echinophthiriidae, Synanthropy, Urban sea lions, Non-invasive sampling

## Abstract

*Antarctophthirus microchir* is a sucking louse species belonging to the family Echinophthiriidae and has been reported to parasitize all species of the subfamily Otariinae, the sea lions. Former studies on this ectoparasite mainly required fixation, immobilization, or death of host species and especially examinations of adult male sea lions are still very rare. Between March and May 2018, adult individuals of a unique “urban” bachelor group of South American sea lions (*Otaria flavescens*) living directly in the city of Valdivia, Chile, were studied regarding their ectoparasite infestation status. For first time, a non-invasive method in the form of a lice comb screwed on a telescopic rod and grounded with adhesive tape was used for sample taking process. Overall, during combing different stages of *A. microchir* were detected in 4/5 *O. flavescens* individuals, especially at the junction between the back and hind flippers. Our findings represent the first report of *A. microchir* infesting individuals of this synanthropic colony and fulfilling complete life cycle in a sea lion group despite inhabiting freshwater and in absence of females/pups. Our “telescopic lice comb apparatus” offers a new strategy to collect different stages of ectoparasites and a range of epidermal material, such as fur coat hair and superficial skin tissue for a broad spectrum of research fields in wildlife sciences in an unmolested and stress reduced manner.

**Electronic supplementary material:**

The online version of this article (10.1007/s00436-019-06273-2) contains supplementary material, which is available to authorized users.

## Introduction

*Antarctophthirus microchir* is a member of the family Echinophthiriidae belonging to the suborder Anoplura, the sucking lice (Durden and Musser 1994; Leonardi and Palma 2013), which represents a group of permanent, obligate, and wing-free ectoparasitic insects of eutherian mammals exhibiting a hematophagous lifestyle (Kim and Ludwig 1978). Representatives of Echinophthiriidae are of special interest since they are the only sucking lice family which infest host species with semi-aquatic lifestyles, such as Pinnipedia and American river otters (*Lontra canadensis*) (Murray 1976; Kim 1985; Leonardi and Palma 2013). The host range of the genus *Antarctophthirus* comprises different pinnipeds including the families Odobenidae, Otariidae, and Phocidae (Leonardi and Palma 2013). Compared with the other six host-specific members within the genus, *A. microchir* infests a broader range of host species. Thus, findings on this parasite were reported from all six species of the otariid subfamily Otariinae, the sea lions, which show a wide geographical distribution and occur in both hemispheres (Kim et al. 1975; Leonardi and Palma 2013; Leonardi et al. 2014). Previous findings reported on *A. microchir* from free-ranging South American sea lion (*Otaria flavescens*) pups from Argentina and included re-description (Leonardi et al. 2009), morphological studies (Leonardi et al. 2012a) and numerous publications on biology (Leonardi et al. 2012b; Leonardi and Lazzari 2014), transmission (Leonardi et al. 2013), and population dynamics (Aznar et al. 2009) of this parasite. First description of *A. microchir* parasitizing *O. flavescens* in Chile was published in 2008 (Crovetto et al. 2008).

The South American sea lion shows a wide geographical occurrence commonly ranging along the Pacific coast of Peru and Chile, the Atlantic coast of Southern Brazil, Uruguay, Argentina, and on the Falkland Islands (Vaz-Ferreira 1982). These polygynous marine mammals live in distinct social structures including harems or bachelor colonies (Cárdenas-Alayza 2017). The Chilean city Valdivia is located approximately 15 km east of the Pacific Ocean and since the late 1970s this city has been harboring a synanthropic bachelor group of *O. flavescens* (Schlatter 1976)*—*a unique “urban” colony of sea lions, which represents the only colony worldwide permanently living in a freshwater habitat. To date, this sea lion colony is composed of approximately 70 individuals between 2 and 15 years of age which still exchange with the Pacific colonies. Their daily presence at the local fish market, a hot-spot of interaction between humans, sea lions, stray dogs, cats, and birds, leads to a remarkable close contact between sea lions and inhabitants or domestic pets within the city of Valdivia. At the same time, this close contact enables transmission of different pathogens. Consistently, a parasitological investigation on the endoparasite fauna of this sea lion colony was carried out and several protozoan and metazoan taxa bearing zoonotic potential [i.e., *Cryptosporidium, Giardia, Neobalantidium* (former *Balantidium*; Mathison and Pritt 2019), Diphyllobothriidae gen. sp., Anisakidae gen. sp.) were detected (Hermosilla et al. 2016a).

In past decades, most investigations on echinophthiriid lice required capturing and fixation techniques (Murray and Nicholls 1965; Kim 1972; Kim 1975; Thompson et al. 1998; Mehlhorn et al. 2002; Crovetto et al. 2008; Leonardi et al. 2012b), immobilization applying anesthetic protocols (Murray and Nicholls 1965; Thompson et al. 1998; Dailey et al. 2005; Leonardi et al. 2014, 2016), or even death of free-ranging pinniped host species (Scherf 1963; Murray and Nicholls 1965; Murray et al. 1965; Kim 1972). Furthermore, several investigations were performed during necropsies of found carcasses of *O. flavescens* individuals (Morgades et al. 2006; Gomez-Puerta and Gonzales-Viera 2015).

For the first time, the current study delivers data on the ectoparasite fauna of the synanthropic colony of *O. flavescens* from Valdivia, Chile, by using a self-designed “telescopic lice comb apparatus” for non-invasive lice/nits sample collection. This non-invasive technique is also applicable for future monitoring projects on ectoparasite infestations of other pinniped species [e.g., harbor seals (*Phoca vitulina*) and gray seals (*Halichoerus grypus*)] and might serve to acquire a wider range of valuable biological skin material (i.e., hair, dandruffs, epidermal cells/debris, and ectoparasites) without considerable disturbance.

## Material and methods

### Study area

During March and May 2018, an “urban” bachelor group of South American sea lions (*O. flavescens*) was evaluated within the Chilean city Valdivia for its ectoparasite infestation status. The study area comprised different resting spots of this colony at riverside piers and construction sites (39° 48′ 37.566″ S, 73° 14′ 49.289″ W), the local fish market (39° 48′ 46.84″S, 73° 14′ 54.212″ W), and a swimming platform (39° 48′ 46.988″ S, 73° 14′ 55.24″ W), all being located along or on the freshwater river Calle-Calle within a vicinity of 4 km^2^.

### Non-invasive combing method

**Fig. 1:**
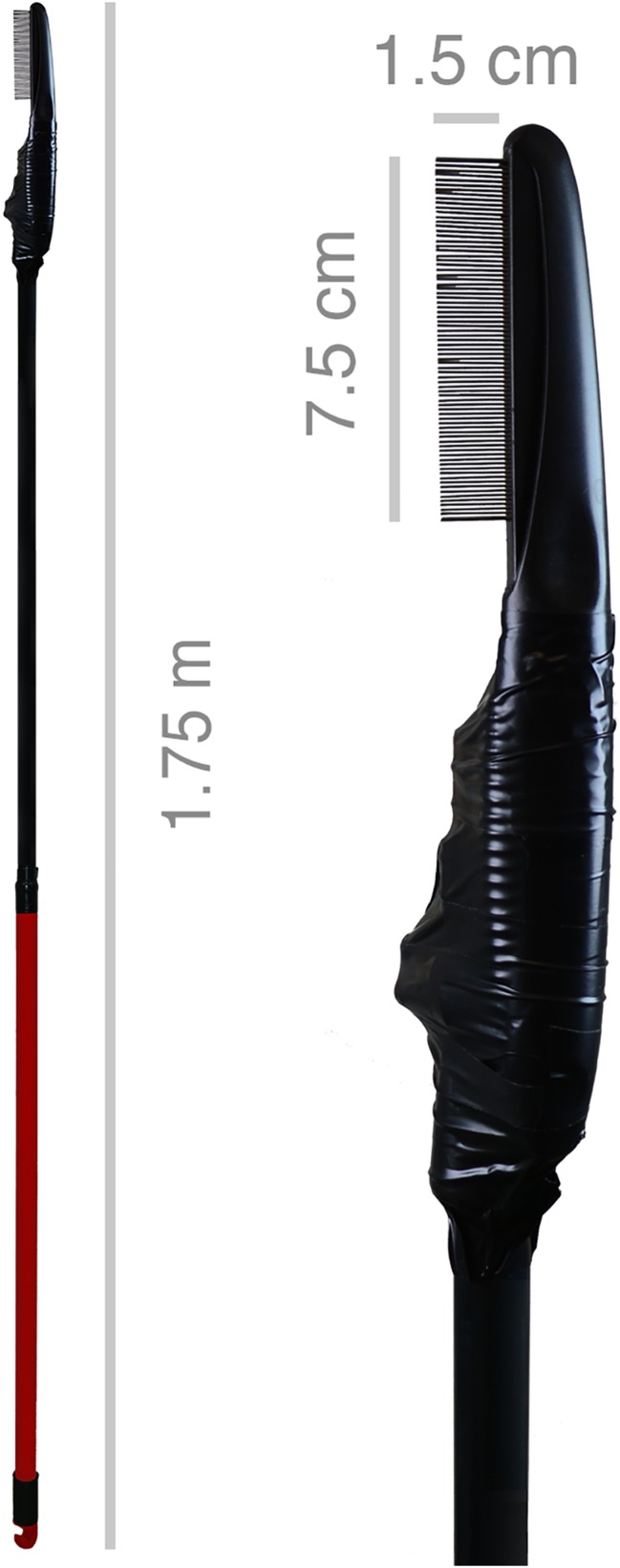
“Telescopic lice comb apparatus”. Overview and close-up of the metal-toothed lice comb on the head of an aluminum telescopic rod fixed with Tesa® tape

In order to distinguish single individuals of the colony and to create a colony register, animals were observed by eye, individually photographed (Sony Alpha 5100®, Minato, Japan; Apple iPhone 8 Plus®, Cupertino, USA) and filmed by a camera drone (DJI Mavic Pro®, Shenzhen, China). For sample collection, a novel non-invasive combing apparatus was here designed: a metal-toothed lice comb, fabricated for domestic pets (TRIXIE® Heimtierbedarf GmbH & Co. KG, Tarp, Germany), bolted on an aluminum telescopic rod fixed with tape (TESA®, Norderstedt, Germany), was used for a broad spectrum of sea lion epidermal material collection (i.e., fur coat hair, lice, nits, and skin tissue samples). The total length of “telescopic lice comb apparatus” was 1.75 m, the metal-teeth row of the comb was 7.5 cm, a single tooth measured 1.5 cm in length and space between each tooth was 0.5 mm (Fig. 1).

### Sample collection procedure

**Fig. 2:**
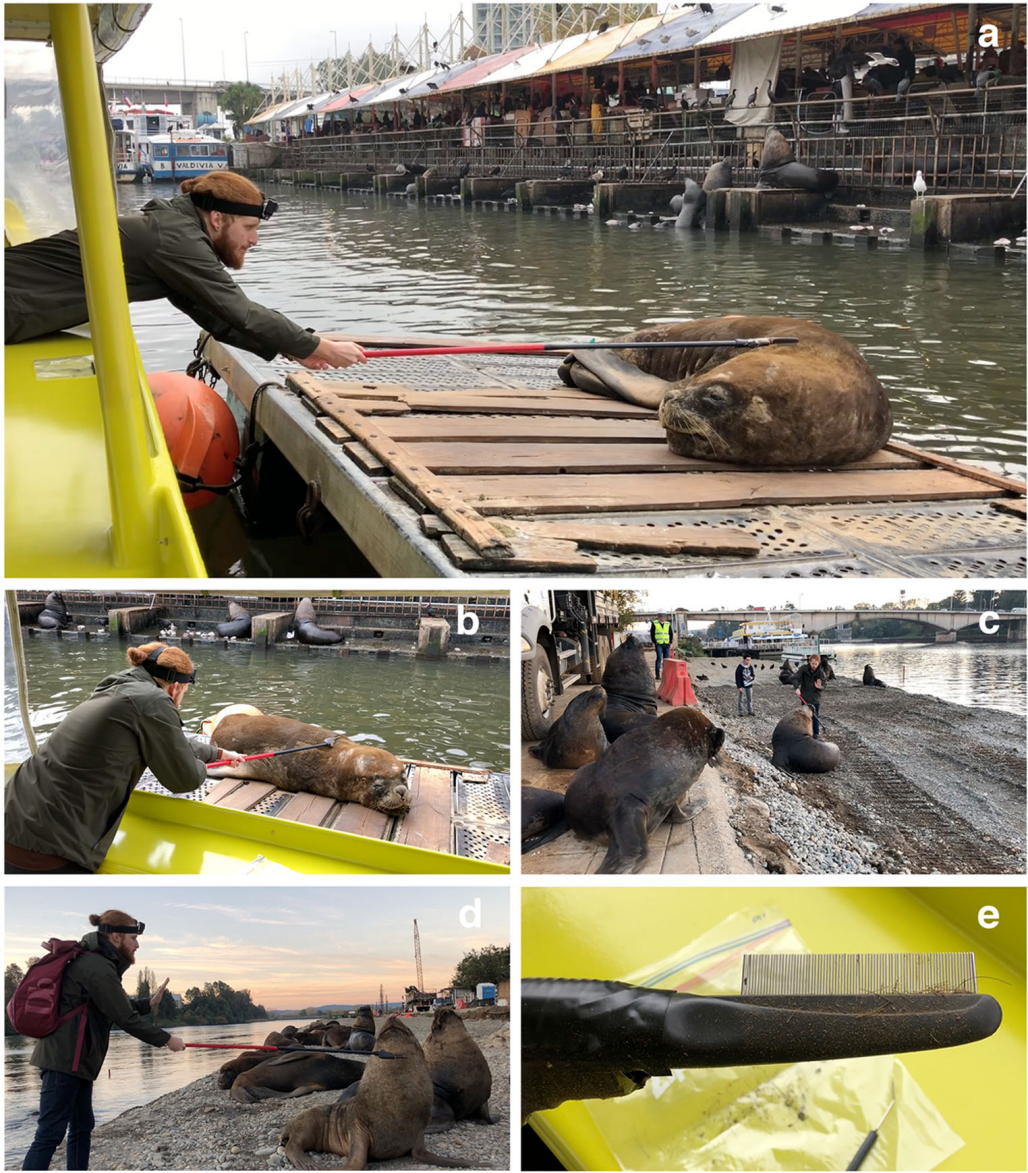
Sampling of the synanthropic *O. flavescens* colony within the city of Valdivia. **a**, **b** Sampling from a boat while the sea lion is resting on a swimming platform on the river of Calle-Calle in front of the local fish market. **c**, **d** Sampling at a typical resting place of *O. flavescens* in Valdivia. **e** Lice comb after combing process, item full of hair, and skin tissue; in the background: dissection needle and ziplock bag for sample storage

**Fig. 3:**
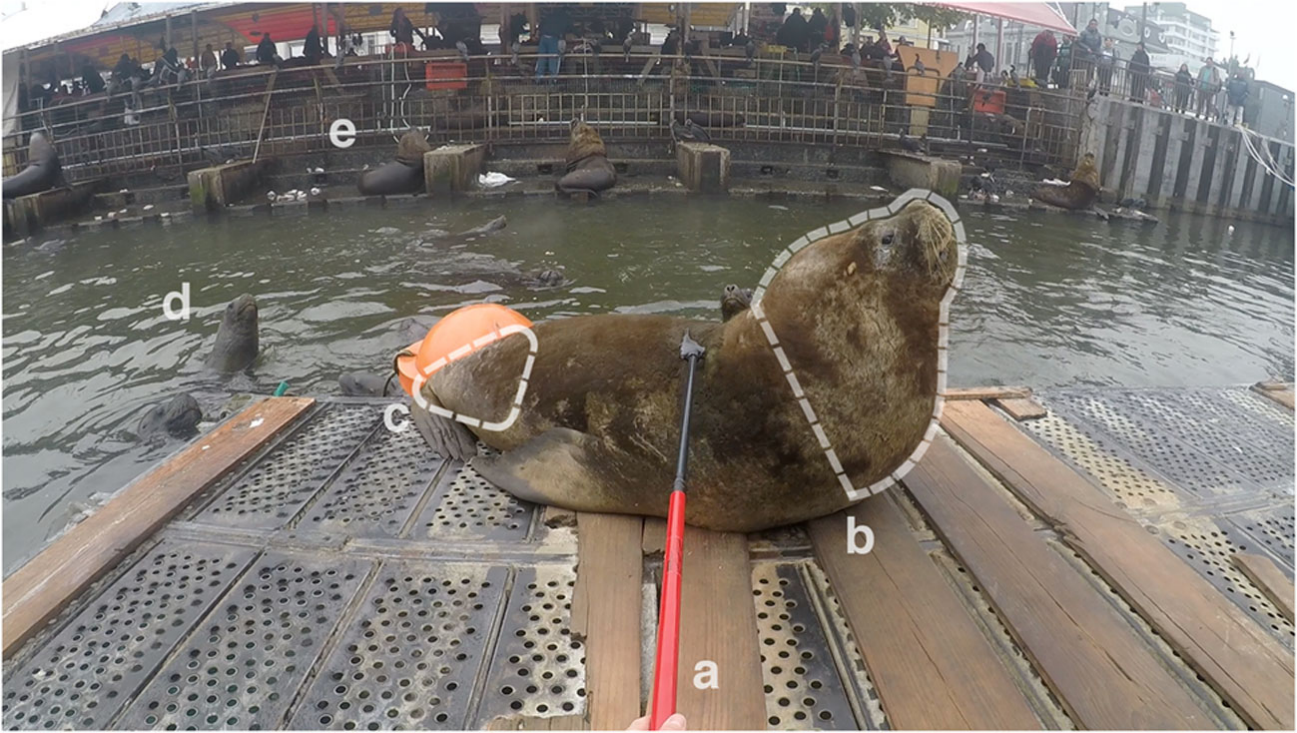
GoPro view: area selection during sampling process on a swimming platform. **a** Application of the “telescopic lice comb apparatus”. **b** Starting point of sampling process: anterior parts of the body to gain sea lion’s trust. **c** Consecutive sampling area: posterior parts of the body. **d** Curious members of the “urban” sea lion colony watching the examination. **e** Local fish market in the background with numerous sea lions waiting for fish waste

For sampling, five non-aggressive sea lions (aged 11 years or older) were chosen on the basis of trustfulness and individual characteristics, i.e., significant skin alterations and apparent pruritus, neoplasia and/or ophthalmic pathologies. When approaching the animals, it was of top priority to radiate a sense of calm and serenity and to maintain direct eye contact with the animals, thereby constantly avoiding frantic movements and loud noises. It appeared beneficial to work from an elevated position or at least to stay at eye-level to the sea lions. Depending on acceptance of individuals to humans, an interspace of at least 0.2–1.5 m was always kept. After selection of an individual, firstly the telescopic rod was carefully moved into the vision field of the sea lion. Secondly, the sea lion was allowed to sniff, lick, and explore the combing tool. Then, cautious initial skin contact was made and the individuals were first combed in anterior parts of their body, especially in regions of the head and neck (Fig. 2a–d). In case of acceptance, posterior parts of the body, dorsum, hind flippers, and pelage were also sampled. Reduced hand movements (e.g., showing palm of the hand) and soft-spoken words clearly showed calming effects on sea lions’ behavior (see Fig. 2c, d). In addition, interactions were documented with a GoPro Hero 5® (San Mateo, USA) camera fixed on a headband (Fig. 3).

Important to note, animals submitted to sampling process were never fed or lured with feed or any other reinforcement auxiliaries. By performing the sampling at open areas near freshwater habitats, the animals always had the chance to leave at any time during the sampling process. Additional video footage of the combing process reveals insights in this special kind of field work (Online Resource 1).


ESM 1(MOV 17856 kb)


Sea lion sampling was conducted in accordance with the Institutional Ethic Commission of the University Austral of Chile (Chile) and to the Justus Liebig University Giessen (Germany). Permission for sea lion sample collection within the city of Valdivia was extended by the Municipality Service of the city of Valdivia and the Chilean National Service of Fishing and Aquaculture (SERNAPESCA) and in accordance with current Chilean Animal Welfare Legislation.

### Storage and morphological identification of *A. microchir* stages

Sucking lice stages were removed from the lice comb by using dissecting needles and transferred to ziplock bags (please see Fig. 2e and Online Resource 2). Thereafter, specimens were preserved in 70% ethanol and analyzed using a light microscope (Olympus CX31®, Shinjuku, Japan). For identification, morphological keys for family and genus level (Murray 1976; Kim 1987; Mehlhorn et al. 2002) and morphometric data of *A. microchir* (Leonardi et al. 2009) were applied.

### Scanning electron microscopy

A female *A. microchir* preserved in 80% ethanol was used for scanning electron microscopy (SEM) analysis. Briefly, the specimen was gently deposited on a circular (10 mm of diameter) glass coverslips (Nunc) pre-coated with poly-_L_-lysine (Sigma-Aldrich). Thereafter, the sample was fixed in 2.5% glutaraldehyde (Merck), post-fixed in 1% osmium tetroxide (Merck), washed in distilled water, dehydrated, dried by CO_2_ treatment, and afterwards sputtered with gold (Villagra-Blanco et al. 2017). The sample was examined by using a Philips XL30® (Amsterdam, Netherlands) scanning microscope equipped with a digital camera allocated at the Institute of Anatomy and Cell Biology (Justus Liebig University Giessen, Germany).

## Results

### Non-invasive combing procedure and microhabitats of *A. microchir* on *O. flavescens* body surface

Starting the combing process at anterior parts of the body, including the forehead, dorsal, and ventral areas of the neck, was necessary to obtain the sea lion’s confidence. All other body areas were studied after cautious skin contact and a phase of familiarization at cranial body parts. Considering this chronological order, selected sea lions tolerated the combing procedure very well and accepted human presence in the middle of the colony (Fig. 2c, d). Recurrent defensive behavior hardly occurred during sampling. Due to natural shyness, younger animals could not be included in the sampling process. The total combing time (i.e., entire body, anterior, and posterior parts) varied between individuals and encompassed between 15 and 45 min/individual and examination. Five sampled sea lions showed clear satisfaction while being combed thereby extending their heads or necks, changing position towards to telescope in order to accommodate certain areas of pruritus to the comb devise (please refer to Online Resource 1).

Overall, all *A. microchir* stages were detected at the caudal back of the animals, especially at the junction between the back and the hind flippers (Fig. 3). The second-stage larva was located near the flank, slightly more ventral than the other stages. No stages of *A. microchir* were found at cranial parts of sea lion’s body even though skin lesions were also apparent at these areas. Collected skin samples consisted of hair, dandruffs, epidermal debris, and ectoparasite stages.

### Infestation with *A. microchir*

**Fig. 4:**
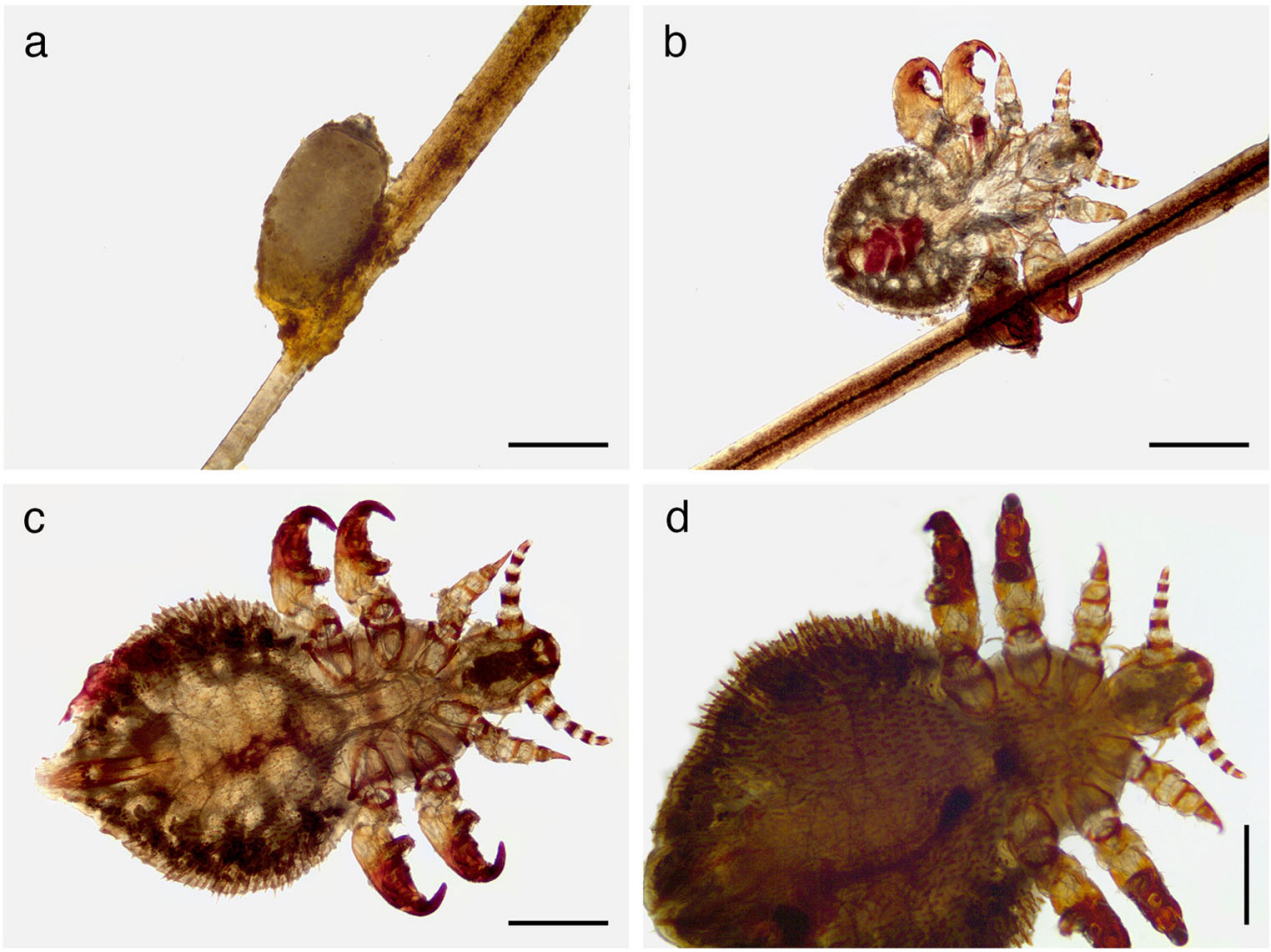
Light microscopic images of different stages of *A. microchir*. **a** Nit. **b** Second-stage larva. **c** Adult male. **d** Adult female. Scale bars, 500 μm

Evaluation of the ectoparasite status of this synanthropic colony revealed a patent infestation with the echinophthiriid louse *A. microchir* in 80% (4/5) of *O. flavescens* individuals*.* In total, nine adult *A. microchir* stages (three females and six males), 1 second-stage larva and three nits of *A. microchir* have been detected (Fig. 4). The presence of all these different stages proved that the entire lifecycle was completed on the animals despite residing in a freshwater habitat and in the absence of females and pups. No other ectoparasite species were here identified.

### Microscopical findings

**Fig. 5:**
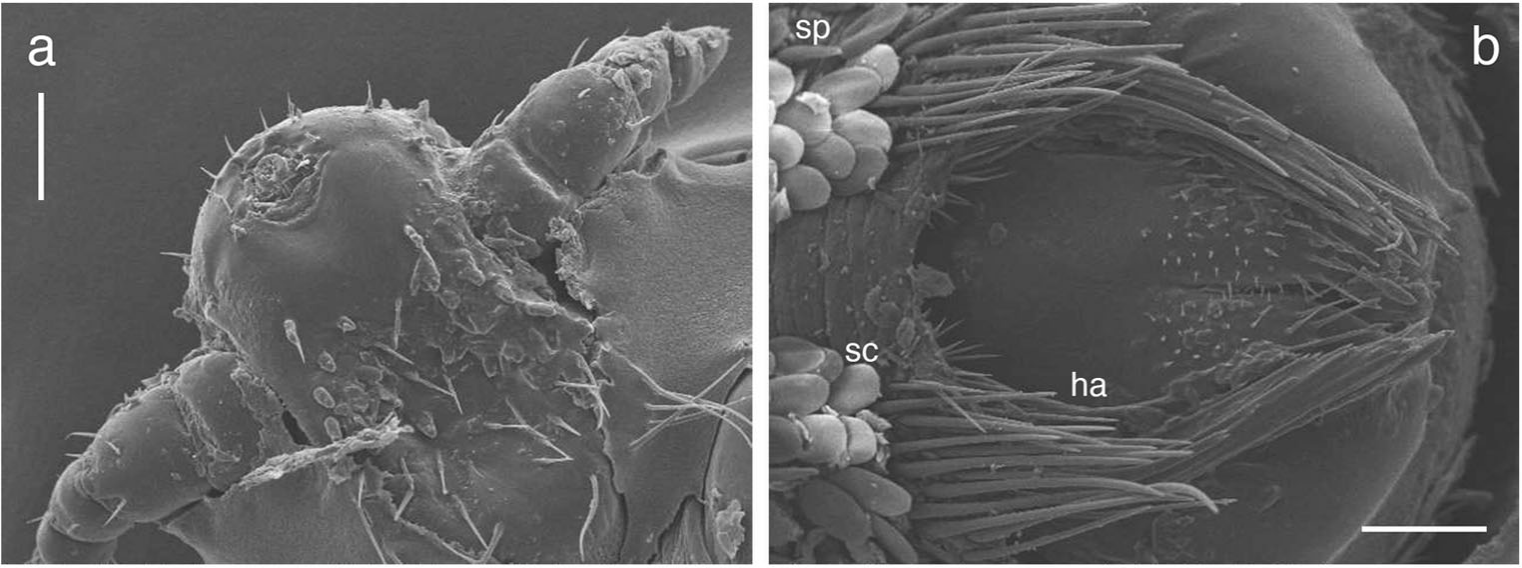
Scanning electron microscopic images of an adult female of *A. microchir.* View of **a** eyeless head and fivefold segmented antennae, **b** female genital porus surrounded by clusters of hairs (ha), leaf-like shaped scales (sc), and spines (sp). Scale bars, 100 μm

Light microscopic examination of different stages (Fig. 4) and SEM analysis of a female specimen (Fig. 5a, b) allowed morphological species identification of *A. microchir* according to literature (Enderlein 1906; Leonardi et al. 2009). The head of *A. microchir* was eyeless (Fig. 5a) and carried thick antennae on both lateral sides. In adult lice, the antennae were composed of five tapering segments (Fig. 4c, d), while the second-stage larva showed antennae with four segments (Fig. 4b). The genital opening of the female stage was laterally bordered with two clusters of hairs, which consisted of long and slightly modified setae (Fig. 5b). Abdominal segments were coated with overlapping scales, which showed a typical flat, oval, and leaf-like shape (Fig. 5b). Spines exhibited a typical elongated shape with a tapering end and could clearly be distinguished from scales (Fig. 5b). Nomenclature of setae was guided by the description of Kim and Ludwig (1978) and additionally according to the revised taxonomy published by Mehlhorn et al. (2002) and Leonardi et al. (2009).

## Discussion

Overall, ectoparasite studies on *A. microchir* in free-ranging South American sea lions generally focused so far on pups due to easier handling while capturing and fixation (Leonardi et al. 2012b; Leonardi 2014), whereas knowledge on infestation status of adult individuals, especially males is still very little (Crovetto et al. 2008). To our best knowledge, the ectoparasite infestation status of free-ranging bachelor colonies of *O. flavescens* has never been examined before.

Taking advantage from a striking adaptation of this “urban” colony of South American sea lions to human activities, we here developed a “telescopic lice comb apparatus” allowing for skin sampling of adult sea lions in a non-invasive and stress reduced way. By obtaining biological material of pinnipeds, e.g., hair (including roots), skin tissue samples (e.g., dandruffs), and different parasite stages, this method opens a broad spectrum of new research fields and could support future projects, including endocrinological (Koren et al. 2002; Sheriff et al. 2011), toxicological (Castellini et al. 2012; McHuron et al. 2012; Peterson et al. 2016), and even molecular genetic analyses of sampled animals. Moreover, this method allows not only unmolested sample collections, but also offers security measures by guaranteeing a certain interspace between researchers and animals. The non-invasive aspect of this method was also reflected by the choice of sampling spots. As such, each animal could interrupt examination and leave at any time the combing process. Since many individuals of this colony were cluttered with skin lesions (e.g., alopecia, hyperkeratosis, pruritus), which could also be caused by bacterial or fungal infections, most animals cooperated very well and even seemed to enjoy the treatment due to pruritus relief. This impression was strengthened by the duration of the combing process, which lasted up to 45 min per individual.

However, despite their excellent adjustment to a life in the city, it has to be kept in mind that the current synanthropic sea lion population still represents wild and free-ranging animals (Schlatter 1976) and younger individuals can still migrate to the Pacific colonies at reproductive ages. Consequently, the sampling process delivers a smaller amount of biological material and is more time consuming than examination of fixed animals (Leonardi 2014) or during necropsies. However, in today’s world, where anthropogenic pressure influences and determines wildlife as intensive as never before, non-invasive or minimally invasive methods should constitute indispensable tools in research of marine mammals (Gales et al. 2009), and it is in our responsibility to develop creative, protecting methods for obtaining a broad range of biological material (Kleinertz et al. 2014; Hermosilla et al. 2015, 2016a, b, 2018a, b).

Morphological characteristics of *A. microchir* were intensively studied in the past, and the morphological identification of the current specimen was in accordance with published determination keys (Enderlein 1906; Murray 1976; Kim 1987; Mehlhorn et al. 2002), the re-description (Leonardi et al. 2009), and SEM examination of this species (Leonardi et al. 2012a).

Regarding predilection sites of the genus *Antarctophthirus*, Kim (1975) reported eyelids, hips, hind flippers, and tail as main body sites of sucking lice. However, Leonardi et al. (2012b) failed to detect *A. microchir* on hind flippers of pups and mainly found them on the back and the belly. Important to note, due to fixation, anterior parts of pup bodies were not examined (Leonardi et al. 2012b). Another study reported *A. microchir* to infest the basis of the snout (Crovetto et al. 2008). In contrast to other monoxenous lice genera, *Antarctophthirus* infests a wide range of host species, involving eared seals (Otariidae), walruses (Odobenidae), and earless seals (Phocidae) (Leonardi and Palma 2013), which may all differ in their mode of locomotion, pelage, and geographical distribution (Leonardi et al. 2012b). As already described for the closely related chewing lice of chicken (Johnson et al. 2012), it was discussed that *Antarctophthirus* infestation of certain areas of hosts may represent a microhabitat selection. Enderlein (1906) suggested that in contrast to *Echinophthirius* spp., which are characterized by the absence of scales and mainly infest areas of seals head, the presence of scales in case of *Antarctophthirus* spp. could be essential for lice breathing activities in case that parts of the body related to respiration (e.g., head, snout) would not be infested. Consistently, in the current study all stages of *A. microchir* were found in posterior parts of the body, especially at caudal parts of the back close to the junction to the hind flippers accordingly to Kim (1975).

Different echinophtiriid lice transmission routes have been reported, whereby all of them can only occur if the pinniped host is ashore (Kim 1975). In case of *Proechinophthirus fluctus* and *A. callorhini*, parasitizing the Northern fur seal (*Callorhinus ursinus*), Kim (1972) proposed direct transmission from cows to pups, especially in first hours after parturition, as the major pathway of transmission. Pup-to-pup transmission of lice may also play a pivotal role, particularly in crowded colonies (Kim 1975). In contrast, horizontal transmission between cows and bulls was described as unlikely due to low lice densities on adult seals (Kim 1975). Indirect transmission through lice/larval stages that have fallen from hosts have not been described so far but might occur as this transmission route is well-known for closely terrestrial lice genera (e.g., *Haematopinus*, *Pediculus*). In line to direct transmission, cow-to-pup transmission was also confirmed as main transmission route for *A. microchir* under natural conditions (Aznar et al. 2009; Leonardi et al. 2013). Early stages of *A. microchir*, i.e., eggs and first larval stages, were reported to mainly occur on pups, which avoid water contact during their first weeks of life (Leonardi and Lazzari 2014). Consistently, in vitro experiments revealed that these lice stages poorly tolerated periods underwater (Leonardi and Lazzari 2014), which is in accordance with weak water resistance of eggs revealed in other studies on echinophtiriid lice (Murray and Nicholls 1965). However, in contrast to first-stage larvae, later larval stages and adults of *A. microchir* tolerate periods of submersion (Leonardi and Lazzari 2014). Interestingly, the synanthropic *O. flavescens* population in Valdivia exclusively consisted of male individuals, with the youngest members being aged approximately 2 years. The fact that nits, second-stage larva, and adult lice (females and males) were detected on these animals, proves that *A. microchir* is capable to fulfill the entire life cycle within this bachelor group even in the absence of pups, yearlings, or females. The ever-granted availability of fish at the local market may shorten or even occasionally cut the hunting periods of these animals thereby resulting in extended periods ashore. Based on the distinct social behaviors of this synanthropic bachelor group, the animals exhibit longer circadian resting periods on platforms, riverside piers, and around the local fish market lying in close contact to each other, which might increase the chance of horizontal lice transmission. Overall, the current data indicate that adult *O. flavescens* males might play a role as reservoir hosts for *A. microchir* even in coastal habitats, where gatherings of bachelor males mainly occur at the borders of the colonies (Cárdenas-Alayza 2017).

This report delivers first insights in *A. microchir* infestation of a synanthropic colony of South American sea lion (*O. flavescens*) males living and evidencing that *A. microchir* is capable to fulfill its life cycle in a freshwater habitat. However, the knowledge on *A. microchir-*borne diseases in free-living adult sea lions is still little and will be expanded in the near future. In the current study, the usefulness of our non-invasive combing method was clearly demonstrated and will allow sampling of biological material (e.g., hair, epithelial cells) from pinnipeds for a broad spectrum of other investigations.

## Electronic supplementary material

ESM 2 (MP4 7207 kb)
